# A comparison of the effects of oral vs. intravenous hydration on subclinical acute kidney injury in living kidney donors: a protocol of a randomised controlled trial

**DOI:** 10.1186/s12882-017-0447-3

**Published:** 2017-01-19

**Authors:** Shona Mackinnon, Emma Aitken, Ryan Ghita, Marc Clancy

**Affiliations:** 1 Department of Renal Transplantation, Queen Elizabeth University Hospital, 1345 Govan Road, Glasgow, G51 4TF UK; 2 College of Medical, Veterinary and Life Sciences, Wolfson Medical School Building, University of Glasgow, University Avenue, Glasgow, G12 8QQ UK

**Keywords:** Acute kidney injury, Hemodynamics, Kidney transplantation, Laparoscopy, Lipocalins, Living donors, Nephrectomy

## Abstract

**Background:**

Optimal treatment for established renal failure is living donor kidney transplantation. However this pathway exposes healthy individuals to significant reduction in nephron mass via major surgical procedure. Laparoscopic donor nephrectomy is now the most common method for live donor transplantation, reducing both donor post-operative pain and recovery time. However this procedure exposes kidneys to additional haemodynamic stresses. It has been suggested that donor hydration—particularly the use of preoperative intravenous fluids—may counteract these stresses, reducing subclinical acute kidney injury and ultimately improving long-term renal function. This may be important in both preservation of donor renal function and recipient graft longevity.

**Methods/Design:**

A prospective single-centre single-blinded randomized controlled trial will be carried out to determine the effects of donor preoperative intravenous fluids. The primary outcome is donor subclinical acute kidney injury (defined as plasma NGAL, >153 ng/ml) on day 1 postoperatively. Secondary outcomes include intraoperative haemodynamics, recipient subclinical acute kidney injury, perioperative complications and donor sleep quality.

Donors will be randomised into two groups: the intervention group will receive active pre-hydration consisting of three litres of intravenous Hartmann’s solution between midnight and 8 am before morning kidney donation, while the control group will not receive this. Both groups will receive unlimited oral fluids until midnight, as is routine. Plasma NGAL will be measured at pre-specified perioperative time points, intraoperative haemodynamic data will be collected using non-invasive cardiac output monitoring and clinical notes will be used to obtain demographic and clinical data. The researcher will be blinded to the donor fluid hydration status. Blinded statistical analysis will be performed on an intention-to-treat basis. A prospective power calculation estimates a required sample size of 86 patients.

**Discussion:**

This study will provide important data, as there is currently little evidence about the use of donor preoperative fluids in laparoscopic nephrectomy. It is hoped that the results obtained will guide future clinical practice.

**Trial registration:**

This study has been approved by the West of Scotland Research Ethics Committee 3 (reference no. 14/WS/1160, 27 January 2015) and is registered with the International Standard Randomised Controlled Trial Number Register (reference no. ISRCTN10199225, 20 April 2015).

## Background

### Chronic Kidney Disease and Renal Transplant

Chronic kidney disease (CKD) has an estimated prevalence of 8.5% in the UK adult population [1]. A small but significant proportion of patients develop end stage renal disease (ESRD) [2]—irreversibly reduced renal function where renal replacement therapy, either dialysis or transplant, is required for survival [3–5]. Currently around 4800 patients in Scotland are on some form of renal replacement therapy, with over 50% of these patients having received a renal transplant [6].

Renal transplantation is associated with increased life expectancy and quality of life compared to dialysis and so should be considered in all patients with ESRD [7, 8]. However, there is an intense kidney shortage (the average UK renal transplant waiting time for a cadaveric kidney is 3–4 years [9]). Almost 300 people die in the UK each year while waiting for a kidney transplant, and many more are removed from the waiting list, having become too ill for a transplant [10]. Therefore, everything possible must be done to maximise renal transplant benefit, particularly the benefit of living donation which is associated with superior graft and patient survival [11].

The price of better recipient outcomes with living kidney donation is some associated additional donor risk [12]. Earlier studies suggested that kidney donation does not affect mortality [13] but a recent prospective observational study by Mjøen et al. [14], comparing kidney donors with carefully matched controls and longer follow-up periods found kidney donation to be associated with a 30% increase in the relative risk of all-cause mortality over a long follow up period (more than 20 years). Furthermore, Muzaale et al. [15] estimated the lifetime risk of ESRD to be 0.9% in living kidney donors, compared with 0.14% in healthy non-donors. Ethically everything possible must be done to preserve renal function in this group, who are undertaking these risks for no personal health benefit.

### Pneumoperitoneum, Renal Perfusion and Subclinical Acute Kidney Injury

First carried out in 1995, laparoscopic nephrectomy is now widespread and has resulted in increasing live donor transplantation [16]. Despite reducing post-operative pain and speeding up recovery, the technique exposes the kidney to theoretical additional haemodynamic stresses of extremes of posture for optimal access and pneumoperitoneum [17].

The effect of increased intra-abdominal pressure is well characterized in abdominal compartment syndrome—organ dysfunction secondary to intra-abdominal hypertension, defined as ≥12 mm Hg [18]. Pneumoperitoneum usually uses pressures of between 12–15 mmHg. The kidneys are particularly sensitive to haemodynamic changes [19]. Studies have shown a decrease in renal blood flow and renal function associated with pneumoperitoneum, though as renal function returns to normal afterwards this phenomenon has been deemed of “uncertain clinical significance” [20, 21]. Indeed a recent Cochrane review found no difference in kidney function in open vs laparoscopic nephrectomy at 1 year [22].

This may reflect the fact that the kidneys have a large reserve, with approximately 50% of renal parenchyma compromise needed to see changes in eGFR. Therefore, even if renal damage has taken place, it may not be detectable by these functional measures. There is a new and emerging concept of subclinical acute kidney injury—injury not detected by functional criteria, but through measurement of biomarkers associated with tubular necrosis [23].

### NGAL as a Biomarker of Subclinical Acute Kidney Injury

NGAL is a small 25 kDa protein of the lipocalin family, and was first identified by transcriptome analysis of the murine model, investigating the early induction of gene expression after renal ischaemia. Its expression was found to be upregulated in tubular cells after ischaemic injury [24]. NGAL levels have been shown to increase in both blood and urine in response to subclinical acute kidney injury [23, 25].

In an evaluation of 125 specimens from healthy individuals using the Alere Triage® CardioRenal Panel, it was found that the 95th percentile upper limit was 153 ng/mL. A study to determine the clinical utility of this compared the NGAL level of 494 plasma samples from patients in intensive care with the standard RIFLE criteria for acute kidney injury. AUC was 0.83 and a positive test threshold of 150 ng/mL yielded a sensitivity of 0.86 and specificity of 0.62 [26]. It was therefore shown to be a sensitive marker of disease.

There has been wide variation in reports of the diagnostic accuracy of NGAL, including variation in the specificities reported. This may be in part as a result of the different populations studied. NGAL is also produced by activated neutrophils and therefore may be raised in infection or inflammation [24, 25]. Plasma NGAL levels have been shown to be raised in septic ICU patients without acute kidney injury, and therefore plasma NGAL should be used with caution in this population [27]. As our population of interest is healthy, studies looking at populations outside a critical care setting are likely to be more generalizable to our population.

Other studies, using the point of care Triage^®^ NGAL Device, have demonstrated superior diagnostic accuracies. For example, 2-h plasma NGAL measurement of 120 children who underwent cardiopulmonary by-pass obtained AUC of 0.96 and the same AKI threshold of 150 ng/ml yielded a sensitivity 0.84 and specificity of 0.94 [28]. Several other studies have also confirmed the usefulness of these bedside NGAL assays in the clinical setting [29]. Therefore, conclusions drawn from previous studies support the diagnostic accuracy of this method.

In the living donor population, Yoon et al. demonstrated that perioperative plasma NGAL in living donors correlates with 6 month donor eGFR. This correlation was found to be strongest using plasma NGAL at 1 week post-operatively, but a correlation was found at all time points [30].

### Pilot Data

Pilot data obtained at our study centre involving 20 donors indicate that hand-assisted laparoscopic nephrectomy is associated with significant subclinical acute kidney injury, defined as plasma NGAL >153 ng/ml, and that preoperative fluids may reduce the incidence of this. In a non-randomised pilot series, where fluids were administered as per surgeon preference, there was found to be an incidence of subclinical acute kidney injury of approximately 50% in patients receiving <1000 ml of intravenous fluids compared with a 20% incidence in those receiving >2500 ml of intravenous fluids. An inverse correlation between increase in NGAL and post donation eGFR at 6 weeks was also demonstrated [31].

The risk of subclinical acute kidney injury has been demonstrated by Hasse et al. who analysed pooled data from ten prospective observational studies, involving over 2000 patients with cardiorenal syndrome type 1. Subclinical acute kidney injury detected by NGAL was found to increase the risk of adverse outcomes, such as need for renal replacement therapy, hospital mortality, and duration of hospital stay, even in the absence of a diagnostically significant increase in serum creatinine [32].

Any decrease in subclinical acute kidney injury is therefore likely to be beneficial. In the long term, it may result in a small increase in GFR, which may be beneficial for the remaining donor function, and reduced haemodynamic stress on the donated kidney may potentially result in greater longevity of the allograft. Therefore optimisation of perioperative fluid management is likely to have a protective role.

### Fluids in Transplant Optimization

There is some controversy surrounding perioperative fluid administration, and optimal fluid balance. The risks of restrictive fluid administration include inadequate organ perfusion and post-operative nausea, whereas liberal fluid use may increase the risk of prolonged post-operative ileus, poor wound healing and the potential for fluid overload leading to heart failure [33, 34].

There have been variable findings in this field, with some studies demonstrating benefit from liberal fluid administration [35–37], and others demonstrating either deleterious effects or no difference in outcome [38]. There is some suggestion that in “low-risk” patients undergoing mild to moderate surgery, a high-volume fluid strategy is superior [39].

Our donor study population is a very healthy one, and all perioperative complications will be closely monitored for and reported.

Potential mechanisms behind attenuation of subclinical acute kidney injury using preoperative fluids are that optimal hydration results in increased renal venous and arteriolar filling, preventing collapse and congestion. Also, increased inferior vena cava pressure may prevent reduced venous return during pneumoperitoneum, maintaining cardiac output. These mechanisms may also reduce vasoconstriction caused by activation of the renin-angiotensin-aldosterone system [40].

We have chosen to use Hartmann’s solution (compound sodium lactate) preoperatively. This contains the following electrolyte concentrations: Na^+^131 mmol/L, K^+^ 5 mmol/L, HCO_3_^−^ 29 mmol/L, Cl^−^ 111 mmol/L and Ca^2^ 2 mmol/L [41]. Although there is some concern about the possibility of chloride increasing the incidence of AKI, Hartmann’s solution is considered to be a chloride-restrictive intravenous fluid [42].

There have been few studies of donor perioperative fluids in laparoscopic nephrectomy. Intraoperative fluid restriction was associated with a lower urine output, but no difference in post-operative serum creatinine levels or complications [43]. This may be because hydration after the establishment of pneumoperitoneum is too late to attenuate any haemodynamic effects. Donors given preoperative fluid were shown to be more resistant to the haemodynamic compromise associated with pneumoperitoneum [44].

As discussed, existing studies used functional criteria, such as serum creatinine, which detect only damage extensive enough to cause immediate functional changes, and any subtle effects present may be masked by the overwhelming effect of nephrectomy and subsequent compensatory renal hypertrophy (CRH). There is therefore currently a lack of evidence about preoperative fluids in laparoscopic nephrectomy, and significant variation in clinical practice. This study aims to address this.

## Methods

### Study Aims and Hypothesis

This study hypothesis is that the use of preoperative intravenous Hartmann’s solution will result in a decrease in donor subclinical acute kidney injury, defined as plasma NGAL >153 ng/ml.

The primary aim of this study is to determine whether preoperative intravenous fluids result in a decrease in donor subclinical acute kidney injury. The secondary aims include investigating the effects of fluids on various donor and recipient clinical and biochemical outcomes, listed below.

### Study Design

This study proposes that donors will be randomised to one of two groups: one group will receive active pre-hydration with three litres of intravenous Hartmann’s solution between midnight and 8 am before morning donation with unlimited oral fluid as desired, while the other group will receive just unlimited oral fluids until midnight.

### Primary Outcome

The primary outcome will be the incidence of donor acute kidney injury (as defined by plasma NGAL >153 ng/ml) on day 1 post-operatively.

### Secondary Outcomes

- Day 1 change in donor plasma NGAL from baseline
- Donor renal function (serum creatinine and eGFR) day 1–4, week 6 and 1 year, as is standard procedure
- Donor BNP changes
- Recipient change in plasma NGAL from baseline
- Recipient serum creatinine and eGFR at 6 weeks and 1 year
- Delayed graft function (DGF)—defined as use of dialysis in the first week postoperatively
- 1 year graft and patient survival in recipient
- Donor intraoperative haemodynamics, including blood pressure, heart rate, stroke volume index, cardiac index and systemic vascular resistance index (collected from non-invasive cardiac output monitor).
- NGAL levels in blood obtained from renal vein during retrieval and implantation surgery
- Donor and recipient perioperative mortality
- Donor perioperative fluid balance
- Donor fluid boluses and response to fluid challenge.
- Donor and recipient perioperative complications (including cardiorespiratory complications, time to first bowel motion, infective complications, length of hospital stay, readmission)
- Donor sleep quality, assessed using the Richards–Campbell Sleep Questionnaire [45]

### Inclusion criteria

- Adult patients aged >18 years of age. undergoing live donor hand-assisted laparoscopic nephrectomy eligible to participate
- Consent to participate given
- English-speaking or appropriate translation facilities to allow for consent to be valid
- Laparoscopic nephrectomy taking place at the study site
- Transplant taking place between dates of the trial.

### Exclusion criteria

- Patients undergoing open nephrectomy
- Patient unable or unwilling to consent

### Power Calculation to Determine Sample Size

Based on the observational pilot data discussed above, which demonstrated a 50% incidence of day 1 subclinical acute kidney injury in patients receiving <1000 ml of intravenous fluids and a 20% incidence of acute kidney injury (AKI) in patients receiving >2500 ml of intravenous fluids, we anticipate that 39 patients would be required in each arm to detect a similar magnitude of difference with 80% power and a significance level of 0.05. Allowing for 10% attrition, we intend to recruit a total of 86 patients.

### Patient Recruitment and Consent

**Fig. 1:**
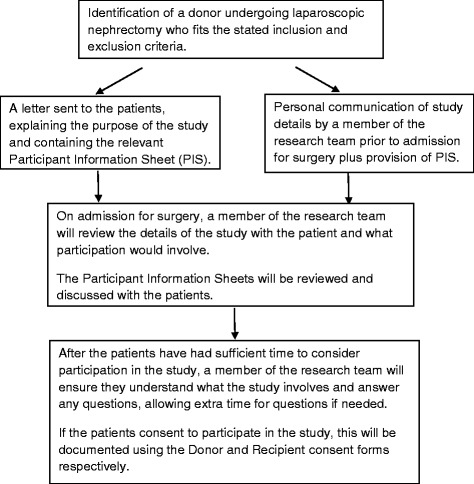
Participant Recruitment Flow Diagram. A diagram showing the ways in which potential participants will be approached and consent will be obtained

Patients who fit the stated inclusion and exclusion criteria will be identified at the time of allocation of a date for surgery. The recruitment process will involve approaching potential participants prior to surgery, and obtaining written consent upon hospital admission [Fig. 1].

### Randomisation and study groups

A computer-generated 1:1 allocation sequence will be created by an independent operator who is not directly involved with the study. Allocation concealment will be achieved using sequentially numbered sealed opaque envelopes. Allocation to treatment arms will be performed by a member of the research team on the day prior to surgery, after the patient has provided valid written consent.

### Blinding

This sort of intervention means that blinding of patients and clinicians is not feasible. Therefore a single-blind approach is being used, where the researcher will be blinded to the fluid hydration status of the patient at the time of data collection.

### Intervention Group

The evening prior to surgery (day-1), between midnight and 8 am, patients in the intervention group will receive three litres of IV Hartmann’s solution. Patients in this group will also be admitted on the evening prior to surgery (day −1) but will not be given intravenous fluids. Both will receive unrestricted oral fluids in line with the European Society of Anaesthesiology perioperative fasting guidelines and will be encouraged to drink freely. It will be ensured that both groups have ready and equal access to unrestricted oral fluids, and any oral and intravenous fluid intake will be recorded via routine fluid charts.

### Pre-operative management

All patients will be admitted on the afternoon prior to surgery as is routine practice and receive entirely standard care in all ways other than the different pre-surgical fluid regimes indicated in this protocol.

### Intra-operative management

All patients will receive standard anaesthetic care and monitoring. Additionally, in order to obtain key haemodynamic measurements, such as cardiac index (CI), systemic vascular resistance index (SVRI) and stroke volume index (SVI) measurements, the use of a non-invasive cardiac output monitor will be required. The NICCOMO^™^ bioimpedance system (Medis, Germany) will be used—a novel and totally non-invasive monitor which employs impedance cardiography to measure cardiac output. It does so by measuring electrical signals produced by changes in the volume and velocity of the blood in the thoracic aorta. This NICCOMO system is complemented by another device—the CNAP^™^ Monitor 500 (CNSystems, Austria). The CNAP Monitor employs the volume-clamp method of Penaz to measure non-invasive arterial blood pressure continuously via two small inflatable cuffs applied to the fingers of one hand. The cardiac output monitor will be attached to the patient shortly after the induction of anaesthesia and will remain in situ throughout the surgical procedure. It will be removed at the end of the operation before the patient is transferred to the recovery room. The anaesthetist will be blinded to the results of the cardiac output monitor.

The cardiovascular response to a single 500 ml fluid challenge will be assessed following the induction to anaesthesia. Cardiovascular parameters with be recorded, then a “stat” 500 ml fluid challenge will be administered and cardiovascular parameters recorded again. Again, the anaesthetist will be blinded to the results of this challenge.

Additionally a single 5 ml sample of venous blood will be obtained from the donor renal vein immediately after retrieval and immediately after implantation to assess the immediate impact on biomarkers of AKI.

### Postoperative management

After surgery, patients will be taken to the recovery room and monitored according to standard hospital policy before return to the transplant ward for post-operative care.

Patients will receive their normal medications prior to surgery unless otherwise directed by the anaesthetist.

### Criteria for discontinuation

Every effort will be made to retain patients in the trial and to minimise withdrawals. Patients with capacity to consent may request to be withdrawn from this study at any time. If the patient develops a severe or life-threatening condition and loses capacity, continued consent will be presumed unless it is deemed that continuation of the trial will result in an adverse outcome for the patient. This is because follow-up of patients who have complications is particularly important for this trial, as it is examining the effect that preoperative intravenous fluid has on these.

In patients who do withdraw from the study, reasons for withdrawal will be documented. All data accumulated up to the time of withdrawal will be retained and included in subsequent statistical analyses.

### Study Centre

The study will be conducted solely within the West of Scotland Renal Transplant Unit. Follow-up will be undertaken in out-patient clinics throughout NHS Greater Glasgow and Clyde by the Renal Transplantation Team.

### Data Collection

Data will be obtained from the ward observation and fluid charts, anaesthetic record, recovery room observation chart, laboratory and bedside blood test results, intraoperative data collection and clinical review in the post-operative period. To avoid any bias, the outcome assessor will be blinded to the fluid hydration that the patient received. The data that will be collected includes demographic data, patient details, perioperative details and primary and secondary outcome data [Table 1].

**Table 1:** Data to be collected

Donor and Recipient Demographic Data	Age Gender Ethnicity Weight Body mass index
Recipient Details	Comorbidities Indication for transplant
Perioperative Details	Volume of donor fluid intake—oral and intravenous Preoperative donor urine output Intraoperative donor urine output Donor fluid boluses and response to fluid challenge. Surgeon performing the operation Duration of surgery Cold and warm ischaemic times
Primary and Secondary Outcome Data	Donor NGAL and BNP (4 measurements): preoperative (day −1), immediately preoperative (day 0), immediately postoperative (day 0), postoperative (day +1) Recipient NGAL (3 measurements): preoperative (baseline), immediately post-operative, postoperative (day +1). Donor eGFR: days 1–4, week 6 and 1 year Recipient eGFR: week 6 and 1 year Recipient delayed graft function (DGF)—defined as use of dialysis in the first week postoperatively 1 year graft and patient survival in recipient Donor intraoperative haemodynamics (including blood pressure, heart rate, stroke volume index, cardiac index and systemic vascular resistance). Biomarkers of AKI, including N-GAL, KIM-1 and leukocyte subsets, in blood obtained from renal vein during retrieval and implantation surgery Donor and recipient perioperative mortality Donor and recipient perioperative complications (including cardiorespiratory complications, time to first bowel motion, infective complications, length of hospital stay, readmission)

### Data Management

Data will be entered on a patient-by-patient basis into a computerised password-protected database on a computer within the Department of Renal Transplantation by an investigator blinded to the study group allocation. Patients will be identified by their study number only. After data analysis has been performed, it will be combined with the randomisation code and the nature of each patient’s perioperative fluid regimen will be revealed. This will be performed in accordance with the Data Protection Act. A case report file will be created for each patient. These will be archived in a locked facility and kept for a period of 3 years.

### Statistical Analysis

Descriptive statistics will be used to describe continuous variables. Results for continuous variables will be reported as mean (+/− standard deviation) or median (interquartile range). The primary outcome, proportion of patients in each group with subclinical acute kidney injury, will be compared using a chi-square test and 95% confidence intervals for the difference in proportions.

If the data for continuous variables follow a Normal distribution, the variables will be compared using a Student’s *t*-test. If data are found to not be Normally distributed, a Mann Whitney *U*-test will be used. Analysis will be performed on an intention-to-treat basis.

Study reporting will be in accordance with the CONSORT guidelines.

## Discussion

As there is currently relatively little data about preoperative fluid use in laparoscopic donor nephrectomy, it is hoped that this study will provide important clinical information. Prior to this study, trials looking at preoperative fluids have shown a beneficial effect on intraoperative haemodynamics. In this study, plasma NGAL will be used to determine whether this effect translates to a reduction in subclinical acute kidney injury, which may be of benefit for both the donor and the recipient.

In terms of the volume of intravenous fluid administration, Mertens zur Borg et al. based the volume of fluid administered on ideal body weight [44]. Though this is a useful approach in terms of monitoring the relative effects of fluid on patient physiology, it was decided that in this study a pragmatic approach to this would be the most feasible option. As approximately 3 L of intravenous fluid has been administered preoperatively previously, it was decided that this volume would be used. This is useful both in terms of facilitating adherence to the protocol and in terms of potential future clinical utility, as it would be more easily reproduced in future clinical practice, should any clinical benefit be found.

It is hoped that, whatever the findings of the study, the results will be of real clinical relevance. If preoperative intravenous fluids are found to be of benefit, then this could lead to their widespread use in clinical practice. If, however, no difference is found between the groups, then live donors may not need be admitted to hospital until the morning of the surgery which would mean greater patient convenience and reduced costs.

## Abbreviations

AKI: Acute kidney injury
CI: Cardiac index
CKD: Chronic kidney disease
CRH: Compensatory renal hypertrophy
DGF: Delayed graft function
eGFR: Estimated glomerular filtration rate
ESRD: End stage renal disease
IAH: Intra-abdominal hypertension
NGAL: Neutrophil-gelatinase associated lipocalin
SVI: Stroke volume index
SVRI: Systemic vascular resistance index
